# Supplementary material for: Analysis of Early Biomarkers Associated with the Development of Critical Respiratory Failure in Coronavirus Disease 2019 (COVID-19)
Source: Diagnostics (Basel). 2022 Jan 28;12(2):339. doi: 10.3390/diagnostics12020339 (PMC8870880; doi:10.3390/diagnostics12020339)
Supplement: Supplementary file 1 [file diagnostics-12-00339-s001.zip › diagnostics-1542577-supplementary.pdf]

Tables S1. Validation analyses of the ROC analyses

**A.** Logistic regression analysis								
			**Univariate**			**Multivariate**		
**Variable**	**Cut off level**	**Odds ratio**	**95%CI**	**P value**	**RR**	**95%CI**	***p* value**	
**Biomarkers at admission**								
CRP (mg/dL)	8.0	44.6	9.1–336.4	<0.001 *	33.2	3.4–874.8	0.007 *	
Interleukin-6 (pg/mL)	133.0	67.5	11.2–596.4	<0.001 *	38.7	2.6–216.1	0.014	
KL-6 (IU/mL)	382.0	28.5	5.1–184.4	<0.001 *	64.6	3.8–2389.5	0.006 *	
**Biomarkers at 3 days after admission**								
Platelet counts (×10^4/uL)	17.2	13.9	2.1–275.9	0.020*	18.6	1.7–572.9	0.033 *	
CRP (mg/dL)	2.2	3.4	0.63–26.1	0.17	0.40	0.010–6.4	0.52	
HMGB-1 (ng/mL)	13.4	34184160.6	0–NA	0.99	1303873456.3	0–NA	0.99	
D-dimmer (ug/mL)	1.9	7.5	1.2–47.2	0.025 *	2.0	0.13–32.0	0.61	
**B**. ROC analysis with 5 repeated 10-fold cross-validation.								
	**AUC**	**Sensitivity**	**Specificity**	**Accuracy**	**Kappa**	**Precision**	**Recall**	**F measure**
**Biomarkers at admission**	0.92	0.99	0.67	0.97	0.65	0.98	0.99	0.99
**Biomarkers at 3 days**	0.88	0.96	0.47	0.92	0.34	0.96	0.96	0.95
**after admission**								

KL-6, Krebs von den Lungen; HMGB-1, high mobility group box 1; ROC, Receiver Operating Characteristic; 95%CI, 95% confidence interval. * A value of $p < 0.05$ was considered to represent statistical significance.

Tables S2. Correlation among biomarkers

**A. Analysis of biomarkers at admission**												
							**R, *p* value**					
	**Neut**	**Lym**	**Plt**	**CRP**	**LDH**	**Fer**	**IL-6**	**IL-18**	**KL-6**	**HMGB-1**	**sCD163**	**D-dimmer**
Neutrophil (uL)	-	0.034	0.19	0.49	0.38	0.30	0.24	0.073	0.25	0.55	0.052	0.32
		0.69	0.024 *	<0.0001 *	<0.0001 *	0.0006 *	0.0077 *	0.44	0.0070 *	<0.0001 *	0.59	0.0001 *
Lymphocyte (uL)	0.034	-	0.019	−0.24	−0.19	−0.12	−0.26	−0.13	0.054	0.15	0.047	−0.17
	0.69		0.025 *	0.0053 *	0.026 *	0.18	0.0037 *	0.16	0.56	0.11	0.62	0.046 *
Platelet (×10^4/uL)	0.19	0.019	-	−0.13	−0.060	−0.20	−0.30	−0.17	−0.087	0.23	−0.10	0.031
	0.024 *	0.025 *		0.13	0.49	0.020 *	0.0006 *	0.076	0.35	0.014 *	0.30	0.72
CRP (mg/dL)	0.49	−0.24	−0.13	-	0.68	0.71	0.64	0.47	0.27	0.17	0.24	0.37
	<0.0001 *	0.0053 *	0.13		<0.0001 *	<0.0001 *	<0.0001 *	<0.0001 *	0.0033 *	0.084	0.012 *	<0.0001
Lactate dehydrogenase (IU/L)	0.38	−0.19	−0.060	0.68	-	0.69	0.53	0.57	0.37	0.31	0.29	0.40
	<0.0001 *	0.026 *	0.49	<0.0001 *		<0.0001 *	<0.0001 *	<0.0001 *	<0.0001 *	0.0011 *	0.0021 *	<0.0001 *
Ferritin (ng/mL)	0.30	−0.12	−0.20	0.71	0.69	-	0.63	0.60	0.31	0.15	0.31	0.21
	0.0006 *	0.18	0.020 *	<0.0001 *	<0.0001 *		<0.0001 *	<0.0001 *	0.0005 *	0.13	0.0009 *	0.020 *
Interleukin-6 (pg/mL)	0.24	−0.26	−0.30	0.64	0.53	0.63	-	0.48	0.21	-0.0049	0.28	0.16
	0.0077 *	0.0037 *	0.0006 *	<0.0001 *	<0.0001 *	<0.0001 *		<0.0001 *	0.021 *	0.96	0.0028 *	0.11
Interleukin-18 (pg/mL)	0.073	−0.13	−0.17	0.47	0.57	0.60	0.48	-	0.13	0.16	0.35	0.16
	0.44	0.16	0.076	<0.0001 *	<0.0001 *	<0.0001 *	<0.0001 *		1.15	0.11	0.0003 *	0.11
KL-6 (IU/mL)	0.25	0.054	−0.087	0.27	0.37	0.31	0.21	0.13	-	0.19	0.28	0.35
	0.0070 *	0.56	0.35	0.0033 *	<0.0001 *	0.0005 *	0.021 *	1.15		0.060 *	0.038 *	<0.0001 *
HMGB-1 (ng/mL)	0.55	0.15	0.23	0.17	0.31	0.15	-0.0049	0.16	0.19	-	0.086	0.11
	<0.0001 *	0.11	0.014 *	0.084	0.0011 *	0.13	0.96	0.11	0.060 *		0.37	0.25
Soluble CD163 (ng/mL)	0.052	0.047	−0.10	0.24	0.29	0.31	0.28	0.35	0.28	0.086	-	
	0.59	0.62	0.30	0.012 *	0.0021 *	0.0009 *	0.0028 *	0.0003 *	0.038 *	0.37		
D-dimer (ug/mL)	0.32	−0.17	0.031	0.37	0.40	0.21	0.16	0.16	0.35	0.11	0.32	-
	0.0001 *	0.046 *	0.72	<0.0001	<0.0001 *	0.020 *	0.11	0.11	<0.0001 *	0.25	0.0007 *	
**B. Analysis of biomarkers at 3 days after admission**												
							**R, *p* value**					
	**Neut**	**Lym**	**Plt**	**CRP**	**LDH**	**Fer**	**IL-6**	**HMGB-1**	**sCD163**	**D-dimmer**		
Neutrophil (uL)	-	−0.19	0.28	0.44	0.42	0.35	0.11	0.69	0.16	0.34		
		0.039 *	0.0021 *	<0.0001 *	<0.0001 *	0.0006 *	0.29	<0.0001 *	0.12	0.0007 *		
Lymphocyte (uL)	−0.19	–	0.29	−0.044	−0.48	−0.45	−0.47	−0.21	−0.17	−0.27		
	0.039 *		0.0011 *	0.63	<0.0001 *	<0.0001 *	<0.0001 *	0.049 *	0.11	0.0080 *		
Platelet (×10^4/uL)	0.28	0,29	–	−0.044	−0.070	−0.092	−0.31	0.079	−0.0088	0.11		
	0.0021 *	0.0011 *		0.63	0.44	0.38	0.0024 *	0.46	0.93	0.28		
CRP (mg/dL)	0.44	−0.044	−0.044	-	0.69	0.67	0.56	0.43	0.36	0.37		
	<0.0001 *	0.63	0.63		<0.0001 *	<0.0001 *	<0.0001 *	<0.0001 *	0.0005 *	0.0002 *		
Lactate dehydrogenase (IU/L)	0.42	−0.48	−0.070	0.69	-	0.77	0.51	0.54	0.42	0.36		
	<0.0001 *	<0.0001 *	0.44	<0.0001 *		<0.0001 *	<0.0001 *	<0.0001 *	<0.0001 *	0.0004 *		
Ferritin (ng/mL)	0.35	−0.45	−0.092	0.67	0.77	-	0.54	0.50	0.45	0.29		
	0.0006 *	<0.0001 *	0.38	<0.0001 *	<0.0001 *		<0.0001 *	<0.0001 *	<0.0001 *	0.013 *		

Interleukin-6 (pg/mL)	0.11	−0.47	−0.31	0.56	0.51	0.54	-	0.19	0.27	0.022		
	0.29	<0.0001 *	0.0024 *	<0.0001 *	<0.0001 *	<0.0001 *		0.073	0.0092 *	0.85		
HMGB-1 (ng/mL)	0.69	−0.21	0.079	0.43	0.54	0.50	0.19	-	0.086	0.27		
	<0.0001 *	0.049 *	0.46	<0.0001 *	<0.0001 *	<0.0001 *	0.073		0.37	0.018 *		
Soluble CD163 (ng/mL)	0.16	−0.17	−0.0088	0.36	0.42	0.45	0.27	0.086	-	0.47		
	0.12	0.11	0.93	0.0005 *	<0.0001 *	<0.0001 *	0.0092 *	0.37		<0.0001 *		
D-dimer (ug/mL)	0.34	−0.27	0.11	0.37	0.36	0.29	0.022	0.27	0.47	-		
	0.0007 *	0.0080 *	0.28	0.0002 *	0.0004 *	0.013 *	0.85	0.018 *	<0.0001 *			

Neut, neutrophil; Lym, lymphocyte; Plt, platelet; LDH, lactate dehydrogenase; Fer, ferritin; IL-6, interleukin-6; IL-18, interleukin-18; sCD163, soluble CD163. * A value of $p < 0.05$ was considered to represent statistical significance.
